# Modulation of growth-related protein expression of native chicken in low altitude in West Jawa, Indonesia

**DOI:** 10.5455/javar.2024.k839

**Published:** 2024-12-27

**Authors:** Andi Mushawwir, Lovita Adriani, Ronnie Permana, Johar Arifin, Renato S. A. Vega

**Affiliations:** 1 Animal Physiology and Biochemistry, Department of Animal Nutrition and Feed Technology, Faculty of Animal Sciences, University of Padjadjaran, Sumedang, Indonesia; 2 Animal Genetics, Department of Animal Production, Faculty of Animal Sciences, University of Padjadjaran, Sumedang, Indonesia; 3 Institute of Animal Science, College of Agriculture and Food Science, University of the Philippines, Laguna, Philippines

**Keywords:** Environmental, growth, metabolism, native chicken, signaling

## Abstract

**Objective:**

The objective of this study was to evaluate the response-ability of local chickens to their rearing habitat, especially at low altitudes, to comprehensively understand the cellular response related to protein growth.

**Materials and Methods:**

Studies of cellular responses related to modulation and growth-related protein salinization for three local Indonesian chicken breeds to low altitudes with high temperatures have been conducted in two locations with altitudes <55 m above sea level. Three hundred local chickens, each consisting of one hundred Sentul, Kedu, and Pelung chickens. During the study, chickens were kept in an intensive system with litter control, equipped with access to play. Blood samples were collected following all standard procedures at the end of the study. Analysis of blood samples has been carried out based on procedures by the protocol on the BioSource KIT and Randox KIT.

**Results:**

Based on the results of the current study, it appears that the overall cellular response of three local chicken breeds shows differences. Sentul chicken and Pelung chicken have better cellular protein expression responses than Kedu chicken. However, when it comes to body weight growth, it seems that protein irradiation to growth is better in Kedu chicken.

**Conclusion:**

The results of the study showed that low altitude with high ambient temperature was better able to be responded to by Kedu chickens, while Sentul and Pelung chickens resulted in both experiencing metabolite shocks, which can be characterized by increased alternative energy provision with creatine phosphate change activity.

## Introduction

Local chickens have the potential to be developed as the main source of animal protein to reduce the dominance of purebred chickens and support national food independence. Currently, 31 chicken groups have specific characteristics and have the opportunity to be used as broilers and layers, including Sentul, Kedu, and Pelung chickens. These three chicken strains are local chicken species that have been widely developed by the community. Sentul chickens have advantages as broilers and layers (dual-purpose). Sentul chicken also has relatively rapid growth compared to other local chickens. Sentul chickens also have the characteristics of faster weight growth and are more resistant to disease. The egg production performance of Sentul chickens is quite good and can produce more than 100 eggs per year [1,2]. Meanwhile, Kedu chicken and Pelung chicken have great potential as local layer and broiler chickens [3,4].

Investigations into local chickens are constantly evolving to find high-rearing efficiency. Kedu chickens and Sentul chickens, as local Indonesian chickens, have higher egg-laying potential than other local chickens. The results of previous studies have widely reported that Kedu chickens have advantages, including the number of eggs in one period of laying [5–7], the results of other studies reported that egg quality showed no difference with other local chicken strains [8–10]. However, the average fertility and hatchability of Kedu chickens appear higher than 90% [11,12]. The results of previous studies that have been reported overall show the superiority of the biological potential of Kedu chickens; however, the efficiency of their ration utilization is not optimal.

Research on the growth and production efficiency of local chickens continues to grow. The genetic factor is one of the important factors determining the growth rate and efficiency of the feed consumed. Local chickens that have been bred and adapted to the tropical environment in Indonesia do not guarantee that local chickens can show optimal performance at various altitudes. It is known that altitude greatly determines the physical factors of the environment, both temperature [6, 8, 11,13], humidity [14–16], and wind speed [13,17]. Research results [18,19] show that local chickens of the same breed appear to show differences in performance with the same altitude or different altitudes. It was reported that chickens generally develop well at temperatures of 23°C–26°C and experience a significant decline in performance at temperatures >28°C [20,21], as well as increased mortality [22,23], bacterial disease infections increased by 45% [24,25].

Many research results show the relationship between temperature and altitude on local chicken performance. The publication reported by [1] shows that not all local Indonesian chickens can adapt well to altitude differences. It was also shown that the increase in global warming seems to have changed the expression pattern of genes related to heat stress, such as the heat shock protein gene [21], and even growth genes [5–7]. The spread and mobility of local chickens to various regions in Indonesia seem to make local Indonesian chickens adapt to compensation for performance that is not optimal. Research results [7] showed that Sentul chickens raised at high altitudes with temperatures <18°C experienced a 15% reduction in growth compared to their place of origin (medium altitude with a 25°C temperature), and the opposite occurred in Pelung chickens [7]. The impact of altitude and temperature has also been shown with hematological conditions [6,7], it was reported that the hematological conditions of local chickens raised at low altitudes showed no difference [26,27]. Another publication showed that the erythrocyte and hemoglobin levels of Sentul and Pelung chickens were not different; both were reared at low altitudes [2,6].

However, the exclusion of hematologic differences, based on a physiologic perspective, does not indicate similarities in the same biological response. It is known that hematologic is the largest component of extracellular fluid [28,29]. Extracellular fluid has a strict homeostasis mechanism [27], this mechanism is strictly maintained even under stress [8]. The hematologic condition cannot be a good indication of performance potential [21], other studies show that specific protein expression is a better indicator to determine the biological potential related to growth [19,20]. Many studies have been reported on the body weight of local chickens reared at low altitudes from various strains, but no publications have shown differences in cellular responses. This cellular response is a manifestation of gene expression, so it is very useful for consideration of the selection process. The point of this study was to find out how well local chickens can adapt to their living environment, especially when they are raised at low elevations and high temperatures.

## Materials and Methods

### Ethical approval

The entire procedure and conduct of this study have been reviewed and confirmed to be acceptable by fulfilling the ethical requirements of animal experimentation by the Ethics Board for Animal Experiments, BATN, with number: 78/An-RE/2/23.

### Animal sample and study location

**Figure 1.**
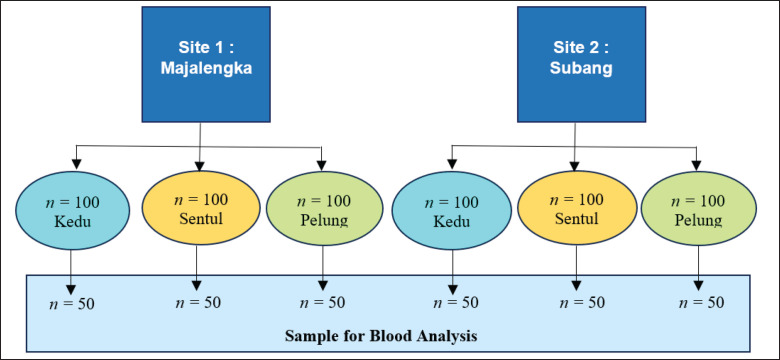
Distribution of chicken samples (n) and number of blood samples in this study.

Three hundred 4–6-week-old local chickens were used in this study, consisting of three strains: Sentul, Kedu, and Pelung. Chickens were intensively reared in two low-altitude locations (Jatiwangi, Majalengka Regency, and Pamanukan, Subang Regency). Each chicken strain consisted of fifty in each location. An overview of the distribution of chicken samples (*n*) and the number of blood samples in this study is shown in Figure 1. Both research sites are located in the lowlands with altitudes of 55 m above sea level and 45 m above sea level for Jatiwangi and Pamanukan, respectively. During the study, daily temperature and humidity were also recorded, with an ­average of 32.5°C; 80%, and 33.7°C; 81% for Jatiwangi and Pamanukan, respectively.

### Feeding and maintenance system

During the study period, the sample chickens were provided with feed and drinking water ad libitum. The composition of the ration consisted of rice bran, ground corn, fish meal, and soybean meal. The crude protein and metabolic energy content of the rations were 17% and 3,200 kcal, respectively. The experimental chickens were intensively reared. A cage with rice husk litter, for each chicken strain measuring 3 × 5 m, was equipped with a field as access to play with a size of 5 × 15 m.

### Blood sampling and data collection

In this study, three hundred 3 ml tubes containing Ethylenediaminetetraacetic acid (EDTA) as an anti-clotting agent were used, as well as 21G syringes. 100 blood samples for Sentul, Kedu, and Sentul chickens, respectively. Blood sampling was done at the end of the study; 50 chickens for each chicken strain were randomly selected, resulting in a total of 100 samples for one chicken strain (Fig. 1). Blood samples were collected by a sterile procedure using alcohol swabs from the pectoralis externa vein, right wing. Each blood sample was collected using one syringe and one tube for each predetermined sample. Blood that had been collected into the tube was immediately flipped so that the sample and EDTA mixed well. EDTA-containing blood samples were put into a bag filled with ice gel. Three hours after all the samples were collected in one location, they were immediately centrifuged to obtain the blood plasma. A mobile centrifuge was used to separate the plasma at 3,500 rpm.

All blood plasma samples were analyzed using the spectrophotometer technique. All data related to protein expression were analyzed based on the procedures and protocols stated in the Elisa KIT Biosource (USA) manual. Data related to protein metabolism were all analyzed based on guidance from KIT Randox (UK).

### Data analysis

Semi-quantitative analysis using Kruskal-Wallis and Mann-Whitney analysis has been used to determine the effect of type response of local chicken strains reared at low altitudes. The best response difference among local chicken strains has been determined by the Whitney analysis method. All of these statistical analyses have used a 95% confidence level, or *a* = 0.05 or 5%. A Microsoft-developed statistical software application, SPSS IBM 2021 version 38.5, has been used in the data testing procedure of this study.

## Results

Local chickens (Sentul, Kedu, and Pelung) reared at low altitudes have shown different responses in three cellular aspects that have been studied in the current research, which are the expression of proteins related to growth signaling, protein correlates of growth hormone receptors (GHRs), and metabolites that indicate metabolic rates in skeletal and liver cells. These cellular responses are described in detail as follows.

### Protein expression

**Figure 2.**
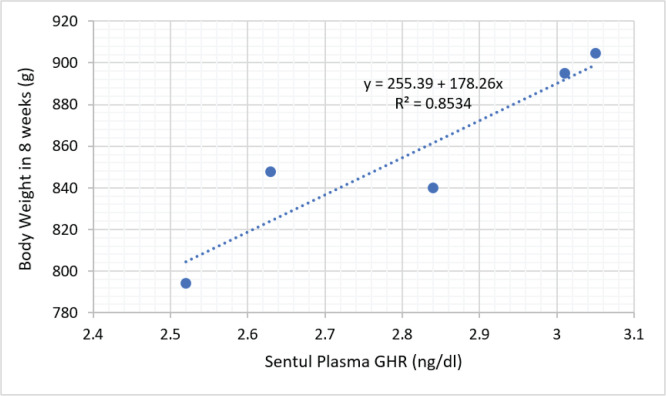
Relationship proportion and prediction model of GHR plasma level with body weight of Sentul chicken.

**Figure 3.**
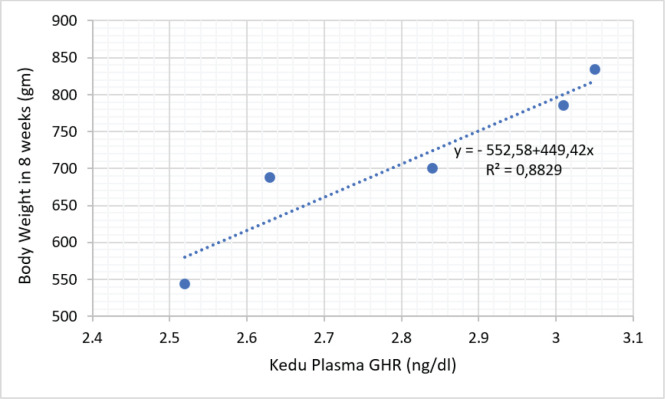
Relationship proportion and prediction model of GHR plasma level with body weight of Kedu chicken.

**Figure 4.**
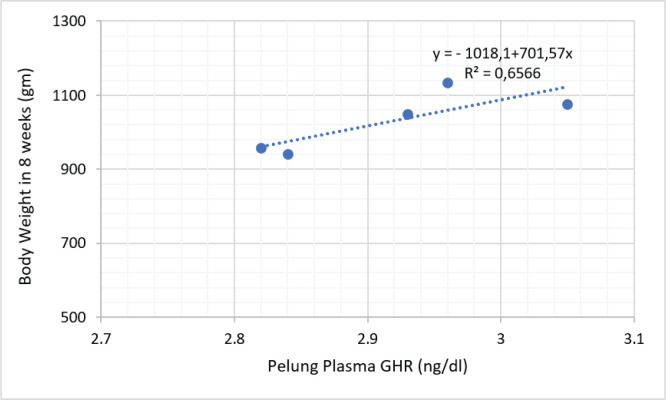
Relationship proportion and prediction model of GHR plasma level with body weight of Pelung chicken.

The results of the cellular protein response study of local chickens reared in low-altitude locations are shown in Table 1. The results of the current study showed that insulin, GHR, and all growth hormone receptor (IGF) variants had higher (*p* < 0.05) concentrations in Sentul and Pelung chickens than in Kedu chickens. It also appears that the concentration of insulin growth factor binding protein (IGFBP)-1–7 in Sentul chicken is not different (*p* > 0.05) compared to Pelung chicken, but the IGFBP level in both chickens is higher (*p* < 0.05) compared to Kedu chicken. Figures 2–4 show the relationship between GHR levels and body weight of Sentul, Kedu, and Opportunity chickens, respectively. It appears that the largest proportion of GHR influence on chicken weight is in Kedu chicken, which is 0.8829% or 88.29%, then Sentul chicken (0.8534% or 85.34%), and the smallest in Pelung chicken, which is 0.6566% or 65.66%.

### Metabolic-related protein metabolism in skeletal muscle

Based on the results of metabolite levels associated with protein metabolism, in Sentul, Kedu, and Pelung chickens showed a significant effect (*p* < 0.05). The levels of these metabolites are shown in Table 2.

**Table 1.** Levels of marker modulators associated with muscle growth regulation in local chickens reared at low altitudes.

Marker (ng/dl)	Types of local chicken strains		
	Sentul	Kedu	Pelung
Insulin	5.42^a^	4.15^b^	5.26^a^
GHR	3.86^a^	2.18^b^	3.08^c^
IGF-1	3.29^a^	2.31^b^	3.05^a^
IGF-2	3.27^a^	3.02^b^	3.52^a^
IGFBP-1	3.19^a^	2.51^b^	3.08^a^
IGFBP-2	2.52^a^	1.06^b^	2.63^a^
IGFBP-3	2.79^a^	1.63^b^	2.63^a^
IGFBP-4	2.58^a^	2.03^b^	3.04^a^
IGFBP-5	2.52^a^	1.24^b^	2.43^a^
IGFBP-7	2.36^a^	0.77^b^	2.13^a^

^a,b^Means followed by different alphabetic superscripts in the same row, indicate significant differences in response (p < 0.05). GHR = growth hormone receptor, IGF = insulin growth factor, IGFBP = insulin growth factor binding protein.

The results (Table 2) show that low altitude with high ambient temperature seems to contribute to the levels of metabolites related to protein metabolism significantly (*p* < 0.05), generally higher than in Kedu chickens. These results suggest that the involvement of hormonal signals in metabolism plays a role in protein metabolism, both through anabolic and catabolic pathways, in response to environmental stress.

## Discussion

One very influential aspect is altitude because it is directly related to the physical factors of the environment, namely temperature and humidity. These two physical factors are two of the most important environmental factors evaluated to get a scientific picture of the interaction between the environment and the genetic factors of the local Indonesian chicken. It is known that the spread of local chickens has received different responses from the community in terms of raising these chickens. The expected maintenance objectives appear diverse, mostly as meat-producing chickens, some as egg producers, and even as the community has cross-bred these local chickens. The pure breeds of local chickens and the results of their crosses also determine their adaptability to temperature.

Previous studies have shown that these two local chicken breeds (Sentul and Pelung) have higher growth [13], and are local chickens with better meat-producing potential [8,30]. An intriguing result was that the levels of insulin and other cellular proteins in Sentul chickens were generally similar (*p* > 0.05) to those in Sentul chickens. Although Sentul chickens have a high growth rate [1,7,26], the impact of high temperatures can be a major obstacle to their anabolism [8].

Physical environmental conditions greatly affect the performance of Sentul, Kedu, and Pelung chickens because breed/strain is also related to physiological and biochemical responses. Heat irradiated mainly to the body of local chickens directly gets a gene response in the body of Sentul chickens [11]. The gene response can determine the rate of catabolism [14,22] and anabolism [4,5,19] in the chicken cells. The genetic ability manifested by the gene response can also determine the performance of the local chicken.

Related to this temperature with the gene response of Sentul, Kedu, and Pelung chickens to the expression of protein metabolism, several important parameters that can be a measure of cellular protein responses related to growth and anabolism are insulin and growth hormone. These two parameters can describe the relationship between protein salinization and growth; several previous studies have shown, for example, its relationship with growth rate [5,15] and increased anabolism [3,5,8,17–20], increased glucose uptake and synthesis of non-essential amino acids [3,11]. Several previous publications have also shown a strong association, enhancing anabolic hormones, especially insulin [19,21]. IGF [5,8] growth hormone (GH) [6] to muscle mass in broilers at normal drumming temperature [27], even with an increase of 30°C from normal temperature [6]. Other publications show the proportion of strong relationships between the body weight growth of ducks and waterfowl to IGF levels in blood plasma [12].

**Table 2.** Metabolites related to protein and energy metabolism in skeletal muscle cells of local chickens raised in the low-altitude.

Metabolic (mg/dl)	Types of local chicken strains		
	Sentul	Kedu	Pelung
Creatinine	2.72^a^	2.06^b^	2.91^c^
Creatinine kinase	0.83^a^	0.64^b^	0.88^a^
Albumin	4.39^a^	3.52^b^	5.43^c^
Uric acid	3.11^a^	2.36^b^	3.35^b^
Glucose 6-phosphate	1.85^a^	1.03^b^	2.21^a^
Total protein	6.62^a^	5.18^b^	7.45^c^
NEFA	2.62^a^	3.63^b^	1.63^c^
TAG	69.39^a^	78.37^a^	58.73^b^

^a,b^Means followed by different alphabetic superscripts in the same row, indicate significant differences in response (p < 0.05). NEFA = non-esterified fatty acid, TAG = tariglyceride.

The phenomenon of the inhibitory effect of high temperature at low altitude can be known through regression analysis between GHR and the body weight of local ­chickens, respectively.

When associated with previous studies, it appears that Sentul chickens have the best potential for body weight growth, compared to Sentul chickens [12], and also to Kedu chickens [18]. Reports from other studies show that the growth and body weight of Sentul chickens are higher than those of Kedu chickens [4,10]. However, when associated with the habit of origin of the three local chickens and the rearing location in this study, the potential to adapt to environmental heat is better in Kedu chickens of ­low-­altitude origin. Although the actual expression of signaling-­related cellular proteins by hormones is not so sensitive in Kedu chickens, the potential to adapt to environmental heat is better in Kedu chickens.

The role of hormones has been demonstrated by several researchers, including that insulin is a potent inhibitor of lipolysis and acts specifically through its signaling to specific proteins such as phosphodiesterase, and the decrease after cycle adenine monophosphate (cAMP) formation is significantly increased [9,14–16]. Currently, it has been shown *in vivo* and *in vitro* that atrial natriuretic peptide has a strong stimulatory effect on lipolysis in adipose tissue [12,17,22]. In contrast to the previously reported effects of catecholamines and insulin, this effect is mediated by a cGMP-dependent pathway, also presumably through protein kinase G, to hormone-sensitive lipase (HSL) phosphorylation. Lipolysis in ruminants [22,30] and almost all animal species is also stimulated by growth hormone [18,21], although its lipolytic action is inhibited, and many publications show that it is not well understood [19], and it is modulated by several signals that have autocrine or paracrine characteristics [7,15], As the reesterification process occurs, glycerol does not appear to be utilized (since glycerol kinase, the enzyme controlling the esterification of non-esterified fatty acid (NEFA) [17] and glycerol into triglyceride (TAG) [18], is not present in adipocytes), it is the rate of glycerol release that has been taken as an index of the rate of lipolysis. However, the validity of this index has been cast into doubt by several recent studies showing that there is a use of glycerol in adipose tissue [22,31], but the rate of use does not exceed approximately 10%–20% of the released glycerol [23,30].

The rate-limiting enzyme of the reaction is HSL. HSL activation is associated with a broad spectrum of insulin [17] and GHR [6,25] hormone signaling. The main hormones involved in the regulation of HSL activity are catecholamines and insulin. Associated with the results of the study, the increased proportion of GH influence may be a mechanism for decreasing insulin levels in high-temperature maintenance. This is why the low role of catecholamines and insulin in stimulating lipolysis through adrenoreceptor activation and inhibiting lipolysis through adrenoreceptor activation [15,26].

Metabolites such as creatinine, albumin, uric acid, and total protein in plasma are mainly substrates that can describe protein metabolic activity. Creatinine is also a precursor for energy formation in skeletal muscle tissue, liver, and striated muscle. In addition, it appears that creatinine may be involved in the regulation of several metabolic processes in the body; for example, creatinine has been shown to control the expression of genes encoding unpaired protein 3 in skeletal muscle [8] and muscle carnitine palmitoyl-transferase 1 in cardiac myocytes [15]. Various factors contribute to the presence of NEFA and TAG in plasma, including feed [12], bioactive substances in the ration [6,21], hormones [15,30], and heat and environmental stress [21,31].

If creatinine is present in plasma in excess, it indicates that creatinine is directly involved in providing energy for tissues [12]. The results of [17] showed that the availability of NEFA for all these processes is regulated mainly by their release from the main deposit of TAG in the body, adipose tissue. NEFAs are liberated from intracellular TAGs by the process of lipolysis and are secreted from adipose cells into the interstitial space and, ultimately, into the circulation. The proportion of NEFA that is solubilized from TAG during lipolysis can be re-esterified and thus form TAG in adipocytes. Consequently, the later release of NEFA from adipose tissue is the result of the contributions of lipolysis and re-esterification of NEFA, since lipolysis, in most physiological situations, dominates the control of NEFA release.

Several recent studies have shown that there is glycerol utilization in adipose tissue [16,17], but the level of use does not exceed about 10%–20% of the released glycerol. The rate-limiting enzyme is HSL. Activation of HSL, by involving specific enzymes, appears to be associated with the assumption that many hormones signal [27,26,27]. Several important types of hormones are involved in this regulation of HSL activity, mainly catecholamines and insulin. Catecholamines stimulate lipolysis through activation of b1 and b2 adrenoreceptors and inhibit lipolysis through activation of a2 adrenoreceptors. HS activation is mediated by a cascade that begins with the incorporation of Gs or Gi proteins and the subsequent generation of cAMP through the adenyl cyclase system and subsequent activation of protein kinase A and phosphorylation of HSL. This activity overall indicates the occurrence of energy availability shocks as a result of environmental heat.

## Conclusion

Based on the results of the current study, it appears that the overall cellular response of the three local chicken breeds shows differences. Sentul chicken and Pelung chicken have better cellular protein expression responses than Kedu chicken. However, when it comes to body weight growth, it seems that protein irradiation is better for growth in Kedu chickens. The results of the study showed that the high-altitude lace with a high ambient temperature was better able to be responded to by Kedu chickens, while Sentul and Pelung chickens made both of them experience metabolite shocks.

## List of abbreviations

cAMP, cycle adenine monophosphate; EDTA, ethylenediaminetetraacetic acid; GH, growth hormone ; GHR, growth hormone receptor; HSL, hormone-sensitive lipase; IGF, insulin growth factor; IGFBP, insulin growth factor binding protein; NEFA, non-esterified fatty acid; TAG, triglyceride.
